# Graphene-Based Materials: Biological and Biomedical Applications

**DOI:** 10.3390/ijms22020672

**Published:** 2021-01-12

**Authors:** Massimiliano Papi

**Affiliations:** 1 Dipartimento di Neuroscienze, Università Cattolica del Sacro Cuore, 00168 Rome, Italy; Massimiliano.Papi@unicatt.it; 2 Fondazione Policlinico Universitario A. Gemelli IRCCS, 00168 Rome, Italy

This editorial aims to summarize the eleven scientific papers published in the Special Issue “Graphene-Based Materials: Biological and Biomedical Applications”.

The very important role of graphene and graphene derivatives in the biomedical field is rapidly growing. In this Special Issue, we aim to illustrate the areas of promise while identifying future challenges. In order to do this, we have invited papers from authors working in different graphene-based biomedical applications. For this reason, the Special Issue is rich in both innovative applications and hot reviews that cover a wide variety of topics of graphene in biomedicine.

There is quite a lot of global discussion on whether graphene is a versatile nano-platform for regenerative medicine and tissue engineering applications. Regarding this topic, Memarian et al. [1] combined carbon with bioceramics to fabricate a 3D-printable scaffold with superior characteristics for bone regeneration, investigating the influence of carbon on cell proliferation and osteogenic differentiation in vitro. This study proposes carbon as an additive for a novel three-dimensional (3D)-printable biocompatible scaffold that could become the key structural material for bone tissue reconstruction. Jagiello et al. [2] demonstrated the impact of graphene-based substrates (both graphene oxide (GO) and reduced graphene oxide (rGO)) on the biological properties of mesenchymal stem/stromal cells, a class of adult multipotent stem cells that possess the ability to differentiate into different cell types of mesodermal origin, including osteoblasts, chondrocytes, and adipocytes. Their results point out that GO scaffolds and rGO scaffolds with a low reduction level exhibit potential applicability as novel, safe, and biocompatible materials for utilization in regenerative medicine. Wierzbicki et al. [3] showed how a graphene oxide scaffold is also able to stimulate myogenic progenitor cell proliferation and the endocrine functions of differentiating cells, and therefore their active participation in the construction of muscle tissue. Yang et al.’s [4] review article explores the potential application of graphene oxide (GO) in the fabrication of electrodes for a retinal prosthesis. This review integrates insights from biological medicine and nanotechnology, with electronic and electrical engineering technological breakthroughs, and aims to highlight innovative objectives in developing biomedical applications for retinal prostheses.

Due to the current antibiotic resistance worldwide, there is an urgent need to find new alternative antibacterial approaches capable of dealing with multidrug-resistant pathogens. Elias et al. [5] investigated a nanotechnological strategy consisting of GO or carbon nanofibers (CNFs) combined with light-emitting diode (LED) irradiation as novel nano-weapons against two clinically relevant Gram-positive multidrug-resistant pathogens: methicillin-resistant *Staphylococcus aureus* (MRSA) and methicillin-resistant *Staphylococcus epidermidis* (MRSE). Neither GO nor the CNFs exhibited cytotoxicity but had high antibacterial activity in direct contact with MRSE and MRSA cells. Furthermore, when the GO or CNFs were illuminated with LED light, the MRSE and MRSA cells lost viability. This combined antimicrobial approach opens up many biomedical research opportunities and provides an enhanced strategy for the prevention and treatment of Gram-positive multidrug-resistant infections.

The creation of graphene-based strategies able to deliver antitumor drugs to tumor cells is of immense importance, but the medical translation of these exciting developments is also crucial. Shan et al. [6] describes an efficient nuclear-targeting delivery system prepared from trans-activating transcriptional activator (TAT) peptide-functionalized graphene nanocarriers. The high tumor-targeting capability of the resulting nanocarrier was realized by the strong affinity between TAT and the nuclei of cancer cells, along with the enhanced permeability and retention (EPR) effect of two-dimensional graphene nanosheets. Subsequently, a common antitumor drug, mitomycin C (MMC), was covalently linked to the TAT-functionalized graphene (TG) to form a nuclear-targeted nano-drug MMC-TG. Their results suggest that the as-synthesized MMC-TG is a promising nuclear-target nano-drug for resolution of tumorous metastasis issues at the headstream. Graphene quantum dots (GQDs) usually constitute a single to tens of layers of graphene and are of a size less than a few tens of nanometers. Owing to their exceptional properties, in particular biocompatibility, GQDs are considered a novel material for oncological applications since they are capable of crossing the blood–brain barrier, the barrier that reduces cancer therapy efficacy. Perini et al [7] showed that surface chemistry modulates the GQDs’ biocompatibility. When used in combination with the chemotherapeutic drug doxorubicin, the GQDs exerted a synergistic effect on the tumor cells, but not on the neurons. This appears to be mediated by the modification of membrane permeability induced by the surface of GQDs. These findings highlight that GQDs can be adopted as a suitable delivery and therapeutic strategy for the treatment of glioblastoma, by both directly destabilizing the cell membrane and indirectly increasing the efficacy of chemotherapeutic drugs. In Perini et al.’s [8] review, the authors deeply discuss the current state of the art of GQDs in biomedicine, with a focus on the neuro-oncology field. This review explains how GQDs are becoming great candidates for bio-imaging and neuroimaging, as well as for theranostic and drug delivery applications, especially to the brain. As bio-imaging markers, GQDs are currently being employed both in vitro and in vivo, thanks to their great optical properties as well as good biocompatibility.

In the general context of bio–nano interactions, Domi et al [9] studied the ability of commercial monolayer graphene oxide (GO) and graphene oxide nanocolloids (GOC) to interact with different unicellular systems and biomolecules. The authors analyzed the response of human alveolar carcinoma epithelial cells, yeast, and bacteria to the presence of different nanoparticle concentrations, and by studying the binding affinity of different microbial enzymes. The reported results highlight the variability that can exist in terms of toxicological potential and binding affinity, depending on the target organism or protein and the selected nanomaterial.

Biosensors and optical devices are key assets when attempting the early diagnosis and monitoring of disease. The recent advances in functional magnetic graphene composites for making very sensitive and selective biosensor devices are vividly described in the Li et al. review [10]. Due to the enhanced electronic properties and the synergistic effect of magnetic nanomaterials and graphene, MGCs could be used to realize more efficient sensors, such as chemical, biological, and electronic sensors, compared to their single component alone. The authors describe how graphene has been used in recent years to implement various applications, ranging from physical device construction to sensor development and cancer theranostics. In the review of Zhang et al. [11], a series of new optical graphene-based devices, showing excellent performance and broad application, are reported. The recent research progress of polarizers, sensors, modulators, and detectors that are based on the polarization characteristics of graphene is reviewed. In particular, the polarization dependence effect and broadband absorption enhancement of graphene under a total reflection structure are emphasized, providing a new direction for research of graphene polarization devices.
